# Retrospect of the Medical Sciences

**Published:** 1843-01-21

**Authors:** 


### ENGORGEMENT OF THE UTERUS.

In a pamphlet published by Doctor Clement Ollivier, of Angers, on the treatment of prolapsus uteri, he speaks strongly against the use of differently shaped pessaries, which are employed indiscriminately, without paying attention to the cause of the prolapsus, which, according to Dr. Ollivier, is nothing more than an engorgement. Thence arise the symptoms which are constantly observed, and which are attributed to any cause other than the presence of a foreign body, and its contact with a painful and inflamed surface.

M. Ollivier considers that one of the most frequent causes of this affection in young girls, with whom it is very rare, is masturbation. He says, that one of the most frequent causes of chronic engorgement of the uterus in virgins, or women who do not have any communication with men, is masturbation, which, by gradually inducing disorder in the uterine functions, gives rise at first to spasm of the organ, which affects the secretion of the menstrua; on the other hand, this excitement, if frequently repeated, finally brings on a more or less intense sanguineous congestion, which gives rise to a kind of impermeability of the uterine parenchima, caused by a slight inflammatory affection; then the dysmenorrhœa, at a later period, becoming habitual, induces amenorrhœa, which ultimately determines more dangerous diseases. Sterility is always an inevitable result, unless the diseased state of the uterus being arrested, allows those portions of the viscus which continue healthy to perform their functions; the catamenia may then reappear, but are almost always accompanied by uterine colics; the matrix may recover its powers of conception, but during gestation a period arrives when the uterus, not being able to enlarge freely, on account of the inflammatory action it has undergone before conception, reacts upon the product it contains, and almost always determines an abortion; in this way the pregnancies of women affected with morbid conditions of the uterus almost always terminate.

Masturbation, in causing a disordered condition of the entire uterus, produces more frequently an engorgement of the body of the organ rather than of the neck, whilst an exactly contrary condition obtains in women who have connection with men. In virgins the affection of the body of the uterus is more frequently found, that of the cervix uteri more rarely.

M. Ollivier mentions, among other causes of engorgement of the uterus, the irritation of the sexual organs by primary connection, a cause of irritation of the organ the more dangerous, that it has hitherto escaped the notice of medical men, either because they do not attach sufficient importance to it, or because women conceal from them the knowledge of their illness, notwithstanding the sufferings they endure.

The dysmenorrhœa, which almost always follows abortions, is the result of an inflammatory engorgement more or less considerable, and susceptible of cure; this engorgement is the cause of the sterility that follows miscarriages. The frequency of these inflammatory engorgements observed by the vulgar has rendered abortions more dangerous in their eyes than a delivery at the full period; when they take place during the first pregnancy, they are the more frequently to be attributed to a too great sensibility of the uterus, as yet unaccustomed to the sensations produced by coition. It is this sensibility which gives rise to consecutive inflammatory symptoms; under other circumstances this uterine sensibility causes the disorders which precede menstruation.

M. Ollivier attributes the sterility which occurs to most women in large towns, after their first and second labors, to a similar cause. The editors of the "Journal de Medicine et de Chirurgie Pratiques" observe, with respect to this opinion, that they agree with M. Ollivier, that the engorgement of the uterus may sometimes prevent conception, but that another cause for this pretended sterility in great towns, and Paris especially, must be sought for. Considerations of a different kind will explain the small number of children found in families, whose pecuniary means are not in just relation with their daily expenses.—*Provincial Medical Journal*, Nov. 19, 1842.

### IMPERFECT LUXATION OF THE RADIUS.

Dr. Goyrand, of Aix, has published, in the "Annales de la Chirurgie Française et Etrangère," two instances of a displacement of the head of the radius, not described in surgical works, but of frequent occurrence among children.

On the 9th of September a little girl, three years old, while walking on an unequal pavement, was nearly falling, when her mother caught her, and kept her up by the right hand. The child immediately screamed out, and could not use the limb; she was brought directly to M. Goyrand. The forearm was flexed to one-fourth its full extent, the hand was in pronation, and dependent, the limb motionless, and there was neither deformity nor swelling at the elbow. Any attempt to bring the hand into supination caused the child to scream. M. Goyrand took the right elbow in his left hand, pressing his thumb at the same time on the anterior face of the head of the radius, and having the child's hand in his own. He then extended the forearm, and exercising rather powerful traction, principally upon the radius, while he carried the hand in supination, then, pushing the head of the radius backwards with the thumb, he suddenly flexed the limb, and the displacement was reduced. The pain the child was suffering from was instantly removed, and the little patient could use the hand to carry a piece of cake, which was given her to her mouth.

The second case so completely resembled this, that it is only mentioned to state that the reduction was as easy and sudden as in the preceding.

In 1837, M. Goyrand published a communication in the "Gazette Medicale," on this injury, from which it appears that it is an incomplete luxation of the superior extremity of the radius, forwards. It is met with only in very young children; it is observed most frequently from the age of eighteen months to three years, when falls are frequent, and to prevent them, children are caught by the hand, or else the child is lifted up by the limb to carry it over the kennel. The upper extremity, thus placed in pronation, supports the whole weight of the body; the weakness of the ligaments and muscles at that age favors the separation of the articular surfaces; a luxation does not take place, and consequently there is not an appreciable change in the shape of the elbow, but the extremity of the radius, separated at first from the small head of the humerus, from the traction exerted on the forearm, is carried forwards by the rapid contraction of the biceps, and there results a change of the articular relations sufficient to explain the appearance of the phenomena by which this injury is generally accompanied.

A significant crackling noise warns the surgeon when the articular surfaces have regained their respective positions. The pain is immediately removed, and the use of the limb is so perfectly restored, as to prevent any necessity foi consecutive treatment. —*Ibid.*

